# Protected and un-protected urban wetlands have similar aquatic macroinvertebrate communities: A case study from the Cape Flats Sand Fynbos region of southern Africa

**DOI:** 10.1371/journal.pone.0233889

**Published:** 2020-05-29

**Authors:** Michelle Blanckenberg, Musa C. Mlambo, Denham Parker, Samuel N. Motitsoe, Cecile Reed

**Affiliations:** 1 Department of Biological Sciences, University of Cape Town, Rondebosch, Cape Town, South Africa; 2 Department of Freshwater Invertebrates, Albany Museum–a Rhodes University Affiliated Research Institute, Makhanda (Grahamstown), South Africa; 3 Department of Zoology and Entomology, Rhodes University, Grahamstown, South Africa; 4 Department of Environmental Affairs, Branch: Fisheries Management Research and Development, Cape Town, South Africa; 5 Centre for Biological Control, Department of Zoology and Entomology, Rhodes University, Makhanda (Grahamstown), South Africa; The University of Sydney, AUSTRALIA

## Abstract

Rapid urbanisation has led to major landscape alterations, affecting aquatic ecosystems’ hydrological and biogeochemical cycles, and biodiversity. Thus, habitat alteration is considered a major driver of aquatic biodiversity loss and related aquatic ecosystem goods and services. This study aimed to investigate and compare aquatic macroinvertebrate richness, diversity and community structure between urban temporary wetlands, located within protected and un-protected areas. The latter were found within an open public space or park with no protection or conservation status, whereas the former were inaccessible to the public and had formal protected, conservation status. We hypothesised that; (1) protected urban wetlands will harbour higher aquatic macroinvertebrate biodiversity (both dry and wet) as compared to un-protected urban wetlands, and (2) that the community composition between the two urban wetlands types will be significantly different. Contrary to our hypothesis, our results revealed no major differences between protected and un-protected urban wetlands, based on the measures investigated (i.e. taxon richness, Shannon-Weiner diversity, Pielou's evenness and community composition) during the dry and wet phase. The only exception was community composition, which revealed significant differences between these urban wetland types. These results suggest that human activities (potential littering and polluting) in the un-protected urban wetlands have not yet resulted in drastic change in macroinvertebrate richness and composition, at least from the dry phase. This suggests a potential for un-protected urban wetlands suffering from minimal human impact to act as important reservoirs of biodiversity and ecosystem services.

## Introduction

Temporary (ephemeral) wetlands are small, shallow, isolated depressions that become inundated after sustained rainfall events and can hold water for a few days, weeks or months before completely drying up again [1–3]. These aquatic systems are an important feature in semi-arid landscapes, providing drinking water for wildlife, and breeding and feeding habitats for both terrestrial and aquatic species [4, 5]. Organisms adapted to living in temporary wetlands have the capacity to deal with the alternating wet and dry phases, thus, resulting in unique and specialised aquatic and semi-aquatic communities not found elsewhere [2, 6]. This allows temporary wetlands to contribute a disproportionately high percentage to regional biodiversity estimates [7]. However, their visual disappearance during the dry seasons make them highly susceptible to habitat destruction. As such, temporary wetlands house one of the highest proportion of endangered organisms than any other freshwater ecosystems [8–10].

The rapid migration of humans from the country side to cities, resulting in the expansion of urban lands is one of the fundamental issues contributing to massive human-driven land-use changes (i.e. urbanisation). The United Nations [11] has projected that by 2050 more than two-thirds of human population will be living in urban areas. This is already true for many countries, especially developed countries, like the United States, where the majority of the population already lives in urban areas as opposed to rural areas [12]. Similarly, developing countries are producing one of the highest rates of urban land expansion, seen in the last few decades [13]. Despite the numerous developmental opportunities brought about by urbanisation, the environmental and biodiversity costs can be fairly high [14–16]. However, that does not necessarily mean cities are not important for biodiversity conservation. On the contrary, studies have shown urban areas can harbour important populations of rare and endangered species just like rural ones [17].

Unlike terrestrial and running waters (i.e. streams and rivers) within urban areas, urban wetlands are under-studied [18] and under-appreciated as an aquatic biodiversity resource even though they support comparable diversity [19]. Studies from across the United Kingdom [18, 20, 21] have reported urban wetlands supporting similar biodiversity patterns compared to non-urban wetlands in terms of aquatic macroinvertebrate diversity and community composition. On the contrary, Johnson *et al*. [22] reported significantly lower taxonomic richness and diversity between urban and non-urban wetlands from Colorado, United States, for both amphibians, aquatic reptiles, and aquatic invertebrates. In South Africa, Harmse and Le Grange [23] showed temporary wetlands in urban surroundings of the city of Johannesburg exhibiting high levels of litter and nutrients concentrations as a result of increasing human activity (mainly recreational) and distance to the nearest human settlements. In this study, we compare biodiversity patterns of urban wetlands in the metropolitan City of Cape Town, South Africa. One set of wetlands was found within an open public space and/or park, and designated as un-protected urban wetland. The other set was within a formally protected, conservation area, thus referred to as protected urban wetland. In the latter area, there was restriction on the public accessing the wetlands, which happened to be the last known locality of the critically endangered Micro frog *Microbatrachella capensis* (Boulenger, 1910) (Pyxicephalidae) [24]. On the other hands, the unprotected wetlands were accessible to public all the time and had no formal conservation and protection status.

The present study aimed to investigate and compare aquatic macroinvertebrate taxa richness, diversity and community structure through hatching soil sediments collected during the dry phase and those sampled during the aquatic wet phase, in five protected urban wetlands (within the Kenilworth Racecourse Conservation Area), against five un-protected urban wetlands (Ottery public park space). We hypothesised that protected urban wetlands will have higher aquatic macroinvertebrate diversity and significantly different community composition than un-protected urban wetlands, based on the view that un-protected urban wetlands tend to be impacted by human activities [23]. The effects of habitat transformation due to the presence of invasive alien plants, mainly, Port Jackson willow *Acacia saligna* (Labill.) H.L.Wendl. (Fabaceae) and kikuyu grass *Pennisetum clandestinum* Hochst. ex Chiov. (Poaceae), have been previously reported to have altered both water chemistry and aquatic macroinvertebrate community structure in wetlands of the region where our study was conducted [25, 26]. By studying both the wet and dry phase, we aimed to gather a holistic view of the temporary wetland aquatic macroinvertebrate community dynamics, which are recognised as highly dynamic and indicative of anthropogenic activities i.e. urbanisation [27]. For the dry phase we collected soil sediments during the dry season and conducted hatching assays in the laboratory, following standard methods [28], and during the aquatic wet phase, microcrustacean and aquatic invertebrates (hereafter referred to as aquatic macroinvertebrates), were collected following standard methods [29].

## Methods

### Ethics statement

Permission for fieldwork in the Kenilworth Racecourse Conservation Area and Ottery Public Park was granted by Rob Slater (Conservation Manager) and City of Cape Town, respectively. A scientific collection permit; No. 0056-AAA008-00065, was granted by Cape Nature, which oversees research infrastructure of the province whether in privately owned land or state land in the province of the Western Cape. At the time of this research, University of Cape Town Ethics Committee did not require ethical clearance for research on invertebrates. This research did not involve capture or handling of animals and therefore did not require approval of animal care and use procedures. The field study did not create effects on endangered or protected species.

### Study site

**Fig 1:**
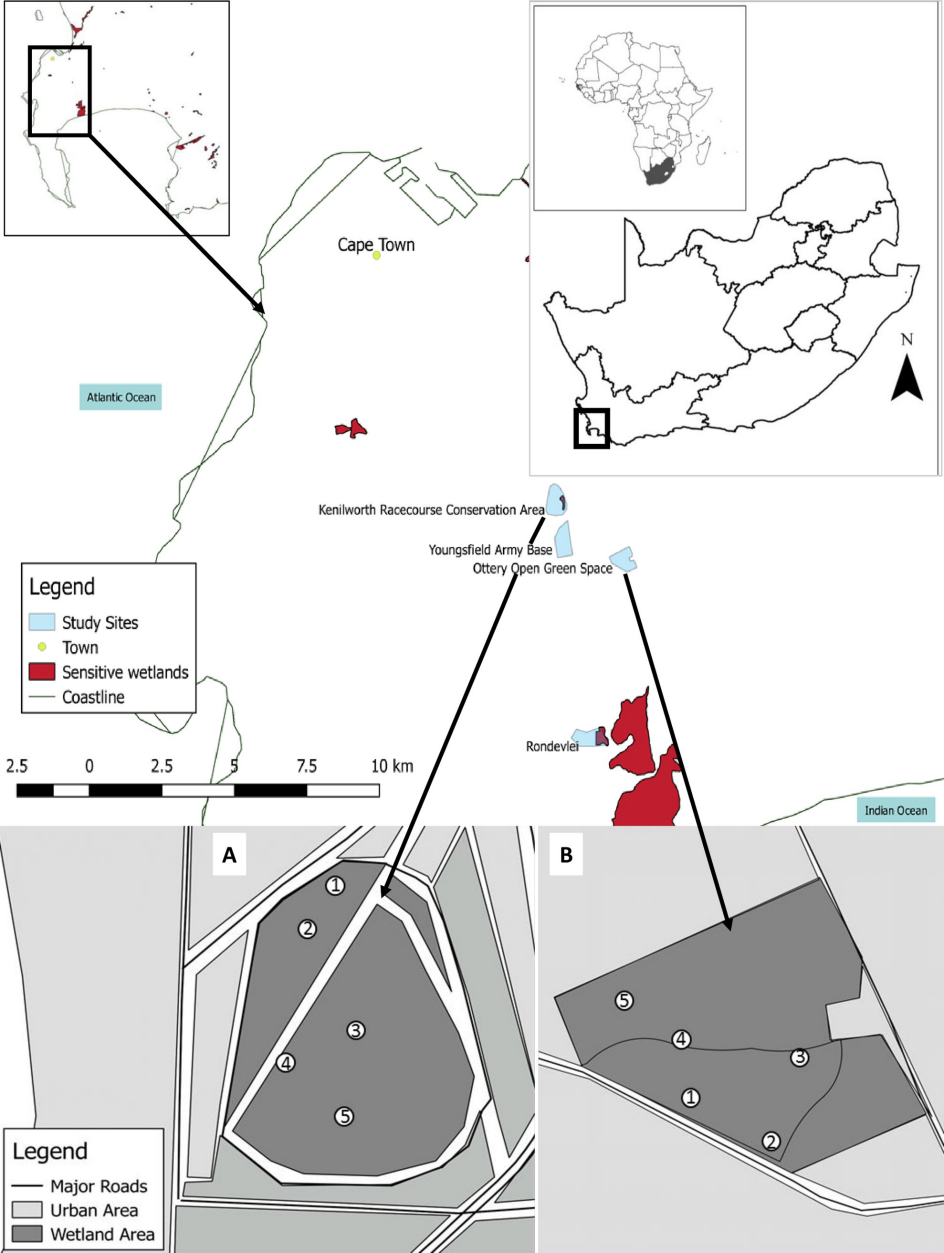
Schematic diagram of the two study areas; a) Kenilworth Race Course Conservation Area and b) Ottery Public Park area, representing protected and unprotected urban wetlands, respectively, and also showing the geographic location of the City of Cape Town and South Africa within the African continent map, all drawn in R-GIS.

To assess the effects of urbanisation on aquatic macroinvertebrates diversity and community structure, ten temporary wetlands were studied in the City of Cape Town, South Africa; five situated within the protected Kenilworth Racecourse Conservation Area (KRCA) and five in the unprotected Ottery Public Park space (Fig 1). The study area falls within the Cape Flats Sand Fynbos region, which is a critically endangered vegetation type found only in the City of Cape Town, southern Africa [30]. Each study area was dominated by different grass species i.e. the mat forming indigenous sedge, *Isolepus rubicunda* (Ness) Kunth (Cyperaceae) in KRCA, while the Ottery Public Park was dominated by an invasive kikuyu grass, *P*.*clandestinum* [25]. These temporal urban wetlands are part of the same wetland class [25], hydrologically isolated, predominately groundwater fed with a significant portion of their inundation received through precipitation, during late winter to summer (late June to November) [26].

The two study areas are within a ~2 km radius to each other (Fig 1). Average conductivity (μS) during the wet phase in KRCA was 2.3 (± 2.2 SD), average temperature (˚C) was 26.9 (± 4.5 SD), average pH was recorded as 5.9 (± 1.0 SD), and average dissolved oxygen (%) 31.9 (± 4.2 SD). Temporary wetlands within the KRCA were relatively restricted from the contemporary urbanisation pressures which included public littering or loitering by wildlife like dogs, goats and sheep, since it a formal conservation area with strict access control. While the Ottery Public Park is currently used as a public recreational area with several walking paths and is frequently disturbed by residents within the surrounding areas, thus experiencing different human-mediated activities as compared to the KRCA sites. However, both areas were, to some extent, subjected to similar pressures happening at regional scale level, like heat island effect, light and noise pollution, contamination due to stormwater runoff and urban invasive species.

### Data collection

#### Dry phase dormant eggs sampling

Soil sediments sampling took place in May 2016, using a standard soil auger with a sharpened iron cutting edge which was used to core the first 10–15 cm of the wetland during dry phase [28, 31]. This ensured that all dormant eggs and tissues of aquatic macroinvertebrates were collected. According to Day *et al*. [32], about 80% of the aquatic macroinvertebrates species present in temporary wetlands are found within the top (i.e. ~ 15 cm) layer of the soil sediment during the dry phase. Three integrated soil sediment samples were collected from 10 urban wetlands, thus making up 30 sampling units (3 samples × 10 wetlands). Soil sediments were collected at three designated regions (right edge, centre and left edge) for each wetland, covering available wetland microhabitats. A total of 30 soil sediment samples (~2 kg) store in a plastic zip-lock bags, were transported to the laboratory and prior hatching assay samples were stored open in a cool and dry temperature controlled room for approximately two months to ensure that the soil was completely dry.

#### Hatching experiments (dry phase)

Hatching assays followed the standard methods described [32, 33], and were conducted in a controlled environment (± 12° C with 24 hours light cycle) at the Department of Biological Sciences, University of Cape Town, South Africa. About 300 g of the dry soil sediments were transferred into a 3 litres non-transparent polyethylene plastic containers (n = 3) and filled with 2 litres of deionised water, sufficient to completely inundate the soil. A total of 30 experimental units (10 sites × 3 regions), where allowed to hatch for a period of 36 days, with aquatic macroinvertebrate hatching recorded after every 24 hour intervals (18 sampling events). In every 24-hour interval, live hatchlings were removed and placed in different containers with inundated soil (non-wetland soil) and fed by adding yeast every 72 hours to allow aquatic macroinvertebrate hatchlings to grow until they could be accurately identified to the lowest possible taxonomic level.

The hatching experiment was conducted in two phases, phase 1, lasted for 26 days to determine hatching success and differences in aquatic macroinvertebrates assemblages and biological diversity. Thereafter, the soil sediments were left to dry naturally for 30 days. Thereafter the second inundation (phase 2) was conducted by re-wetting previously dried experimental units. The second re-wetting (phase 2) lasted for 36 days, and was to account for any viable aquatic macroinvertebrates dormant eggs that prefer multiple re-wetting occasions before they can hatch.

#### Aquatic wet phase macroinvertebrates sampling (wet phase)

Aquatic macroinvertebrates sampling was conducted in September and October 2016 during periods of wetland inundation. The protected wetlands were sampled in both September and October 2016, while the unprotected wetlands were only sampled in September 2016, as wetlands had dried before the October 2016 sampling period. Aquatic macroinvertebrates were collected using a standard square-frame aquatic sweep net with a 235 mm frame and 80 μm mesh size [25, 29]. Similarly, wetlands were divided into three biotopes i.e. submerged vegetation, emergent vegetation and open water. Aquatic macroinvertebrates samples were collected in three designated biotopes separately. Aquatic sweeping followed a standard protocol for sampling temporary and permanent wetlands as described in [29]. Each sweep consists of dragging the net down at 45° angle until nearly touching the bottom, and pulling the net back up at a similar angle to create a sweep arc roughly one metre in length. Aquatic sweep samples were immediately preserved in 10% formalin and stored in a polyethylene sample jars separately. After 48 hours’ samples were transferred into a new polyethylene sample jars and preserved with 70% ethanol solution to prepare for identification and for long-term preservation.

### Aquatic macroinvertebrates identification

Collected aquatic macroinvertebrates were identified and counted using the sub-sampling procedure described in [25]. Briefly, large and rare aquatic macroinvertebrates and microcrustacean taxa (large, easily visible specimens represented by less than 10 individuals per sample) were removed first after observing the sample for the first five minutes. Thereafter, the remaining samples were emptied into a white tray which was divided into grid square cells of equal size, and was randomly sampled to pick out ca. 200 aquatic macroinvertebrate organisms. Following aquatic macroinvertebrates identification and counting, relative aquatic macroinvertebrates abundances were estimated by multiplying observed individual aquatic macroinvertebrates species/genera by the number of grid cells. Thereafter, voucher specimens were removed from the samples and the remaining sample was re-filled with 70% ethanol solution for further microcrustacean identification.

Prior microcrustacean identification and enumeration, samples were allowed to sediment on a stable flat bench surface for a period of 72 hours. Thereafter the ethanol supernatant (~500 ml) was discarded using a top-down siphoning system and care taken not to agitate the sample. About 50 ml of tap water was added, the sample was further homogenised by moderately agitating the sample for five seconds, this was to evenly distribute microcrustacean within the samples. Thus, immediately using a Pasteur pipette 1 ml sub-samples was placed onto a Bogrow tray for microcrustacean enumeration under a dissecting microscope (Leica BS-3300, Magnification 7× ~ 46×). This procedure was repeated per sample until ca. 200 individual microcrustacean counts was achieved (using approximately 20 ml of the sample) [25].

Hatching aquatic macroinvertebrates were collected and identified were possible every 24 hours, and immediately preserved in 70% ethanol solution (voucher specimens), while challenging immature stages transferred to a different microcosm (non-wetland soil and water) and fed yeast to reach maturity for accurate identification. For all phases, aquatic macroinvertebrates were identified to the lowest possible taxonomic level (either genus or species level) using the relevant identification guides and keys [25, 29]. Misidentification of individuals for immature life stages is common, thus majority of microcrustacean identification were done during the last immature instar and where possible (i.e. hatching assay) during the adult life stages. Challenging aquatic macroinvertebrates taxa were send to relevant taxonomy specialist for further identification.

### Statistical analysis

All statistical analyses were conducted in the R programming environment [34]. Species accumulation curves were computed using the ‘vegan’ package (specaccum function), based on the cumulative number of aquatic macroinvertebrates new hatchlings per day, and were used to illustrate experimental sampling effort throughout the study [35]. The average number of aquatic macroinvertebrate taxa hatching during phase 1 and 2 (or 1^st^ and 2^nd^ inundation) between protected and unprotected were tested for normality using the Shapiro-Wilks test (shapiro.test function), and thus data was found to be not normality distributed (Shapiro-Wilks test, p<0.05). To test for significance difference in the number of aquatic macroinvertebrates hatching between treatments i.e. protected vs. unprotected urban wetlands types a non-parametric test, Mann-Whitney U test (wilcox.test function) from the package ‘MASS’ was used.

Additionally, aquatic macroinvertebrates biological diversity indices including taxa richness (S), the Shannon-Weiner diversity index: H’ = -∑ ^*s*^_*i = 1*_ *pi* ln *pi*, (where *pi* is the proportional abundance of taxa *i* in the sample given *s* taxa), and Pielou’s evenness; J*’* = $\frac{H\prime }{ln(S)}$, were computed (using vegan package, see [36]) per sampling occasion (in both protected and unprotected urban wetlands) and inundation phases (i.e. 1^st^ and 2^nd^ inundation) during the aquatic phase and the dry phase respectively. To investigate aquatic macroinvertebrates biological diversity indices response to treatment (protected and unprotected) and inundation (1^st^ and 2^nd^ inundation/wetting) and their interaction, a Linear Mixed-Effects Model using the ‘lme4’ package (lmer function) and post-hoc Tukey test package ‘multcomp’ (glht function) was employed.

Linear Mixed-Effects Models allow the incorporation of fixed factors and random effects that control for correlation in data arising from grouped observations [37], where fixed effects were treatments and inundation phases and sites fitted as random effects.

Additionally, multivariate Regression Trees (MRTs) were used to explore the relationship between treatments and the observed aquatic macroinvertebrate assemblages during the hatching assays using the Bray-Curtis similarity coefficient [38]. The MRT analysis using package ‘mvpart’ [38], species composition of each hatching assays was related to treatment (protected vs. unprotected), wetting (1^st^ vs. 2^nd^ inundation), hatching period (early, intermediate, late) and wetland site (Ottery vs KRCA). Similarly, this was repeated for the aquatic phase sampling relating to treatment (protected vs. unprotected) and month/sampling occasion (October vs September). The percentage contribution of each aquatic macroinvertebrate species (>5% frequency of occurrence) to assemblage composition of each terminal group was calculated. A ‘random forest’ [39] algorithm was applied to estimate the importance of each of the aforementioned variables, allowing for direct comparisons of importance between variables. Permutational analysis of variance (PERMANOVA) using the Adonis function was used to test for significant grouping between treatments, hatching phases and for temporal aquatic macroinvertebrate groupings [40, 41].

## Results

**Fig 2:**
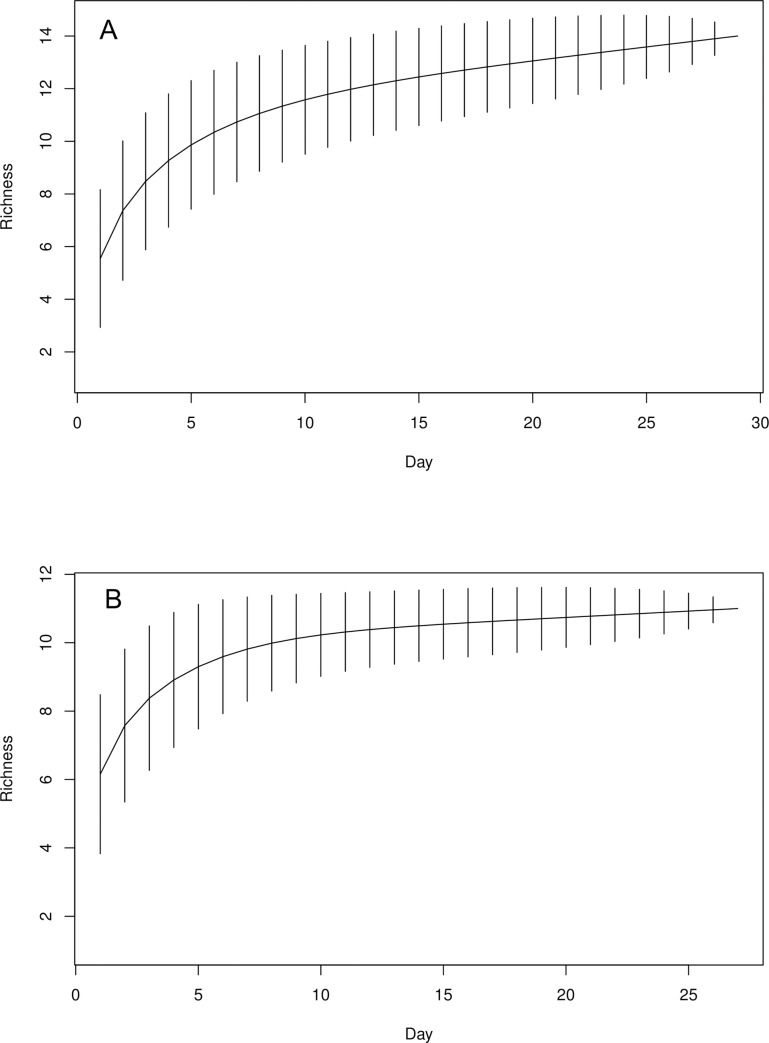
Species accumulation curve showing the number of species hatching per day from soil sediments collected at the; (a) Protected and (b) Unprotected urban wetlands. Expected species richness values (solid line) with 95% confidence internals (vertical lines).

A total of 46 aquatic macroinvertebrate taxa (i.e. 19 hatching from soil sediments and 27 collected during the aquatic wet phase), were collected and identified during the study (Table 1, S1 Appendix and S2 Appendix). During the hatching assays (dry phase), 18 aquatic macroinvertebrates taxa were found in the protected urban wetland sites and only 15 in the unprotected urban wetland sites. The aquatic wet phase yielded more aquatic macroinvertebrates taxa, 27 taxa were collected in the protected urban wetland sites, whereas only 12 were found in the unprotected wetland sites. Three aquatic macroinvertebrates taxa including, *Dugesia* sp. (Turbellaria), *Mesamphisopus* sp. (Isopoda) and *Philonthus* sp. (Coleoptera) were exclusively collected during the hatching assays and not found during the aquatic wet phase sampling. Similarly, *Streptocephalus* sp., *Megafenestra aurita* (Fischer, 1849) and *Lovenula simplex* Kiefer, 1929 were only found during hatching assays in the protected urban wetland sites and *Mesamphisopus* sp. in the unprotected urban wetlands. On the other hand, many more species were observed during the wet phase and not found in dry phase; notably, *Paramelita pinnicornis* Stewart & Griffiths, 1992, *Daphnia barbata* Weltner, 1897, *Daphnia laevis* Birge 1878, *Simocephelus exspinosus* (Koch, 1841) and *Paradiaptomus lamellatus* Sars, 1895. Also, protected urban wetlands had substantial higher number of predatory taxa than the unprotected counterparts during the wet phase. This included several taxa, like dragonfly nymps (e.g. aeshinids, coenagrionids and gomphids), bugs (belostomatids, gerrids, notonectids and pleids) and calanoid copepods found only in protected wetlands (Table 1). Only, a few predatory taxa occurred in both protected and unprotected wetlands; diving beetles (dytiscids) and water boatmen (corixids). The number of hatchlings observed between protected and unprotected urban wetland sites were not significantly different during both the 1^st^ (Mann–Whitney U, W = 105, *p* = 0.30) and 2^nd^ (Mann–Whitney U, W = 177, *p* = 0.64) inundation phases. Both treatments showed a consistent increase in the number of hatchlings, however the unprotected wetlands exhibiting greater variability reached maximum hatchlings and equilibrium at day 15 as compared to day 25 in the protected wetlands (Fig 2). Interestingly, there was no substantial difference in the taxa from the dry phases between the urban wetland types (Table 1).

**Table 1:** Full species list for aquatic macroinvertebrates collected and identified from ten protected and unprotected temporary urban wetlands sites during both the hatching experiment (indicated by asterisk) and aquatic wet phase in May to October 2016, in the City of Cape Town, Western Cape Province of southern Africa.

Class	Order	Family	Taxon	KRCA	Ottery
Arachinida	Hydracarina*			x	x
Branchipoda	Anostraca	*Streptocephalidae*	*Streptocephalus* sp.*	x	
	Cladocera	*Chydoridae*	*Pseudochydorus* (Probably *P*. *gr*. *Globosus)*	x	x
		*Daphniidae*	*Ceriodaphnia*	x	x
			*Daphnia barbata*	x	
			Daphnia pulex^*^	x	x
			*Daphnia laevis*	x	
			Daphnia longispina^*^	x	x
			Megafenestra aurita^*^	x	
			*Simocephelus exspinosus*	x	
		*Macrothricidae*	*Echinisca* sp.	x	x
		*Moinidae*	*Moina* sp.	x	x
Clitellata	Oligocheata	Tubificidae^*^		x	x
Copepoda	Calanoida	*Lovenula*	Lovenula simplex^*^	x	
		*Paradiaptomus*	*Paradiatomus lamellatus*	x	
	Cyclopoida	Cyclopidae^*^		x	x
Collembola	Entomobryomorpha	Isotomidae^*^		x	x
	Poduromorpha	*Onychiuridae*	*Deuteraphorura* sp.*	x	x
		*Hypogastruridae*	*Hypogastrura* sp.*	x	x
Eurotatoria	Ploima	*Brachionidae*		x	x
Gastropoda		*Physidae*	Physa acuta^*^	x	x
		*Planorbidae*	*Ceratophallus* sp.	x	
Insecta	Coleoptera	*Dytiscidae*	*Cybister* sp.	x	x
		Gyrinidae^*^		x	
		*Hydraenidae*		x	x
		*Hydrophilidae*		x	x
		*Hydrochidae*	*Hydrocus* sp.	x	
		*Spercheidae*	*Spercheus* sp.	x	
		*Staphylinidae*	*Philonthus* sp.^*^	x	x
	Diptera	Chironomidae^*^		x	x
		*Culicidae*	*Culex* sp.	x	x
	Ephemeroptera	*Baetidae*	*Cloeon* sp.	x	x
	Hemiptera	*Belostomatidae*	*Appasus* sp.	x	
		*Corixidae*		x	x
		*Gerridae*	*Gerris* sp.	x	
		*Notonectidae*	*Notonecta* sp.	x	
		*Pleidae*	*Plea* sp.	x	
	Odonata—Anisoptera	*Aeshnidae*		x	
		*Coenagrionidae*		x	
		*Gomphidae*		x	
Malacostraca	Amphipoda	*Paramelitidae*	*Paramelita pinnicarnis*	x	
	Isopoda		*Mesamphisopus* sp.*		x
Ologohymenophorea	Peniculida	*Parameciidae*	*Paramecium* sp.*	x	x
Ostracoda	Podocopida	Cyprididae^*^		x	x
Turbellaria	Tricladida	*Dugesiidae*	*Dugesia* sp.*	x	x

**Fig 3:**
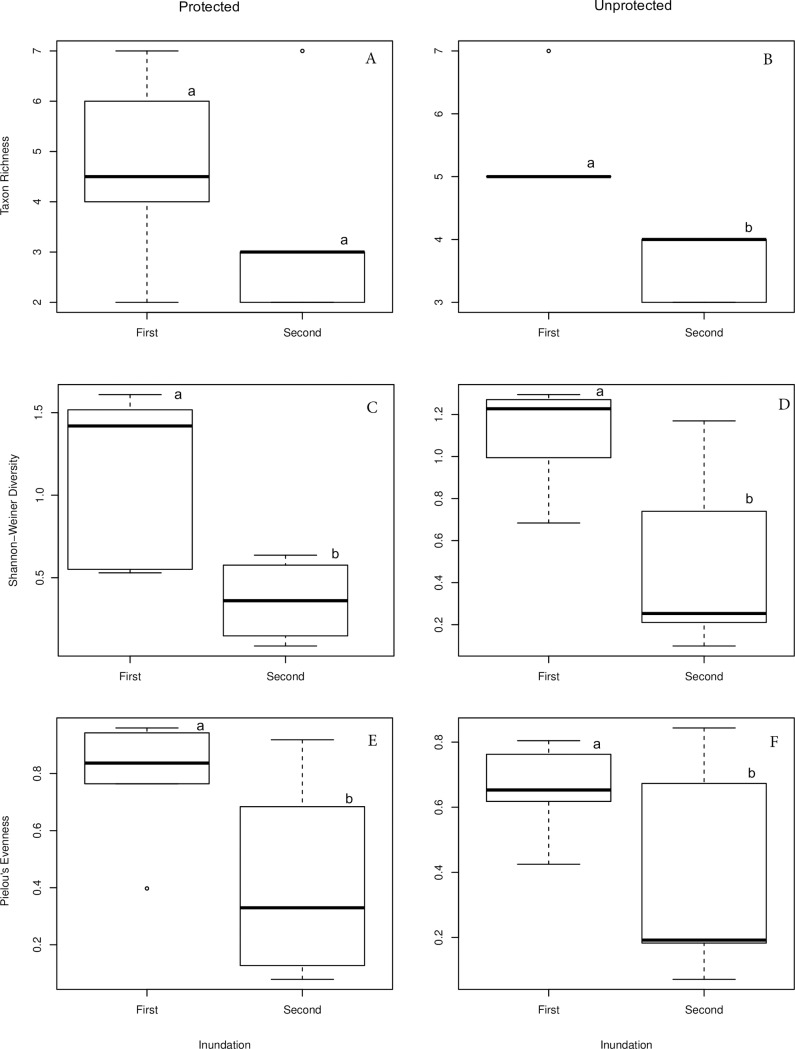
Dry-phase macroinvertebrates biological diversity indices (i.e. Shannon-Weiner diversity, Taxa Richness and Pielou’s Evenness) hatched from dried soil sediments, reporting both the 1^st^ and 2^nd^ inundation phases of the protected vs unprotected urban wetlands. The horizontal black line within the white box represents the median. The white box ranges from the 1st to the 3rd quartile. The upper whisker delimits the 3rd quartile plus 1.5 times the interquartile distance (3rd quartile– 1st quartile). The lower whiskers mark the 1st quartile minus 1.5 times the interquartile distance. Different letters represent significant difference.

Protected and unprotected urban wetlands showed no significant differences in terms of taxon richness, diversity and evenness of macroinvertebrate hatched out from the dry soil sediments, (Table 2). This was true for both the first and second inundation phases. The only cases where there were significant differences was when comparison was made within each wetland type, i.e. comparing the 1^st^ and 2^nd^ inundations of unprotected wetlands (Fig 3). In fact, the 1^st^ inundation phase showed higher taxa richness, diversity and evenness as compared to the 2^nd^ inundation phase.

**Table 2:** LMER results showing the effect of urban wetland type (protected vs. unprotected), hatching inundation (1^st^ and 2^nd^) and the sampling period (September vs. October) on aquatic macroinvertebrates biodiversity indices i.e. taxon richness, Shannon-Weiner diversity and Pielou’s evenness in the City of Cape Town, Westen Cape Province in southern Africa. Significant differences marked with an asterisk.

Predictor	n	Taxon Richness (S)			Shannon-Weiner Diversity (H’)			Pielou’s Evenness (J’)		
		Estimate (± SE)	Z	P	Estimate (± SE)	Z	P	Estimate (± SE)	Z	P
**Dry Phase (Hatching)**										
Protected vs unprotected (1^st^ hatching)	30	0.50 (± 0.63)	0.78	0.43	0.02 (± 0.17)	0.15	0.88	-0.07 (± 0.14)	-0.54	0.58
Protected vs unprotected (2^nd^ hatching)	30	0.48 (± 0.61)	0.76	0.48	0.04 (± 0.19)	0.17	0.86	-0.06 (± 0.12)	-0.50	0.61
Protected (1^st^ vs 2^nd^ inundation)	30	-1.33 (± 1.02)	-1.30	0.19	-0.81 (± 0.22)	-3.61	0.0002*	-0.37 (± 0.12)	-2.94	0.003*
Unprotected (1^st^ vs 2^nd^ inundation)	30	-1.80 (± 0.37)	-4.81	< 0.001*	-0.59 (± 0.18)	-3.24	0.001*	-0.26 (± 0.11)	-2.26	0.02*
**Wet Phase (Aquatic Sampling)**										
Treatment (protected vs. unprotected)	10	-3.25 (± 1.76)	-1.83	0.06	-0.43 (± 0.24)	-1.74	0.08	-0.07 (± 0.08)	-0.98	0.32
Month (Sept vs Oct)	10	-1.25 (± 1.76)	-0.70	0.48	-0.21 (± 0.24)	-0.86	0.39	-0.006 (± 0.03)	-0.21	0.83

**Fig 4:**
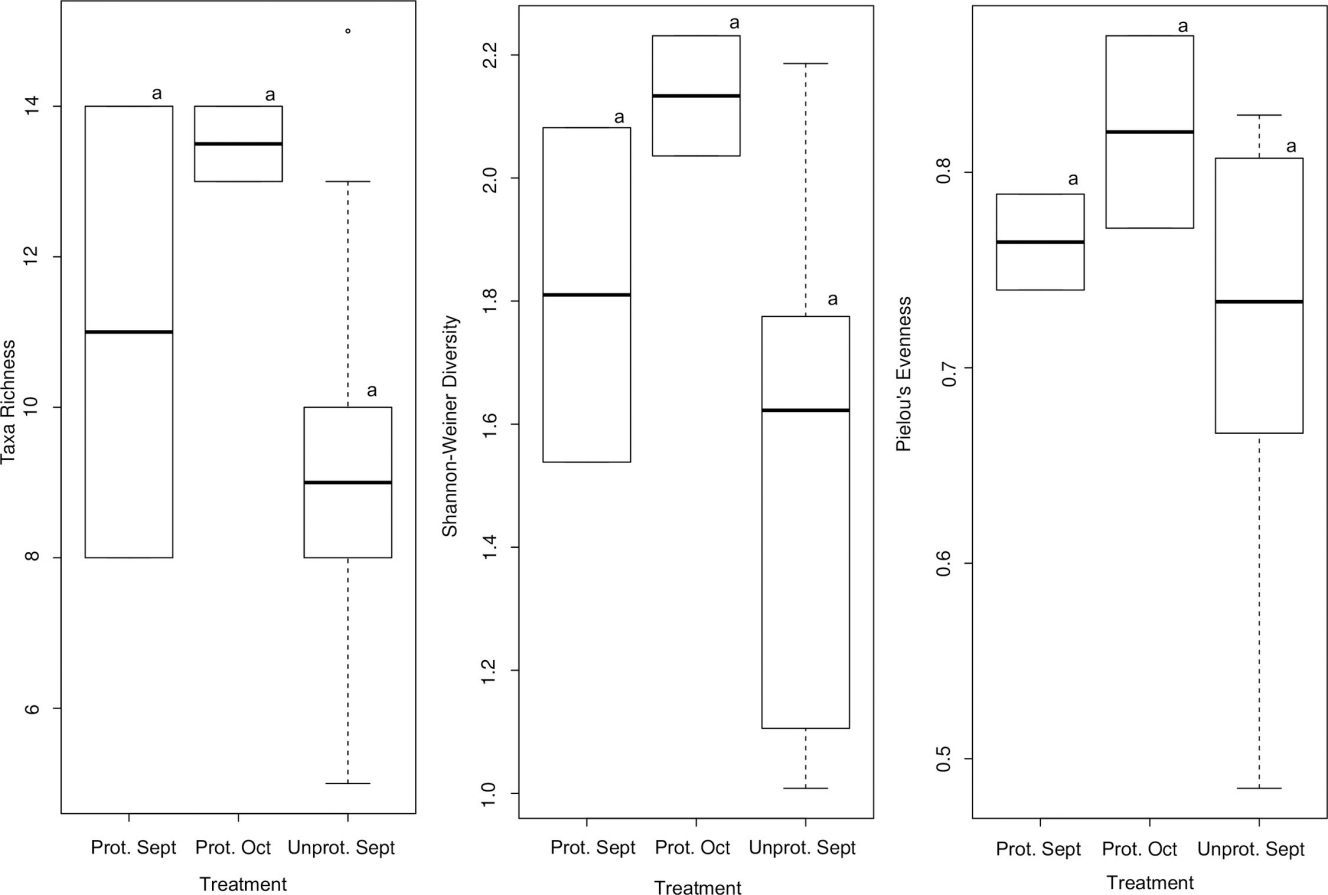
Aquatic macroinvertebrates biological diversity indices (i.e. Shannon-Weiner diversity, Taxa Richness and Pielou’s Evenness) during the aquatic wet phase between the protected (denoted P) and unprotected (denoted UP) wetlands, sampled in September and October 2016. The horizontal black line within the white box represents the median. The white box ranges from the 1st to the 3rd quartile. The upper whisker delimits the 3rd quartile plus 1.5 times the interquartile distance (3rd quartile– 1st quartile). The lower whiskers mark the 1st quartile minus 1.5 times the interquartile distance. Different letters represent significant difference.

Also, during the aquatic wet phase taxa richness, diversity and evenness showed no significant difference between the protected and unprotected urban wetlands (Table 2). However, protected wetland tended to have substantially higher taxa richness and diversity (Fig 4), and non-significant differences are probably due to the great variability observed in samples from unprotected wetland.

**Fig 5:**
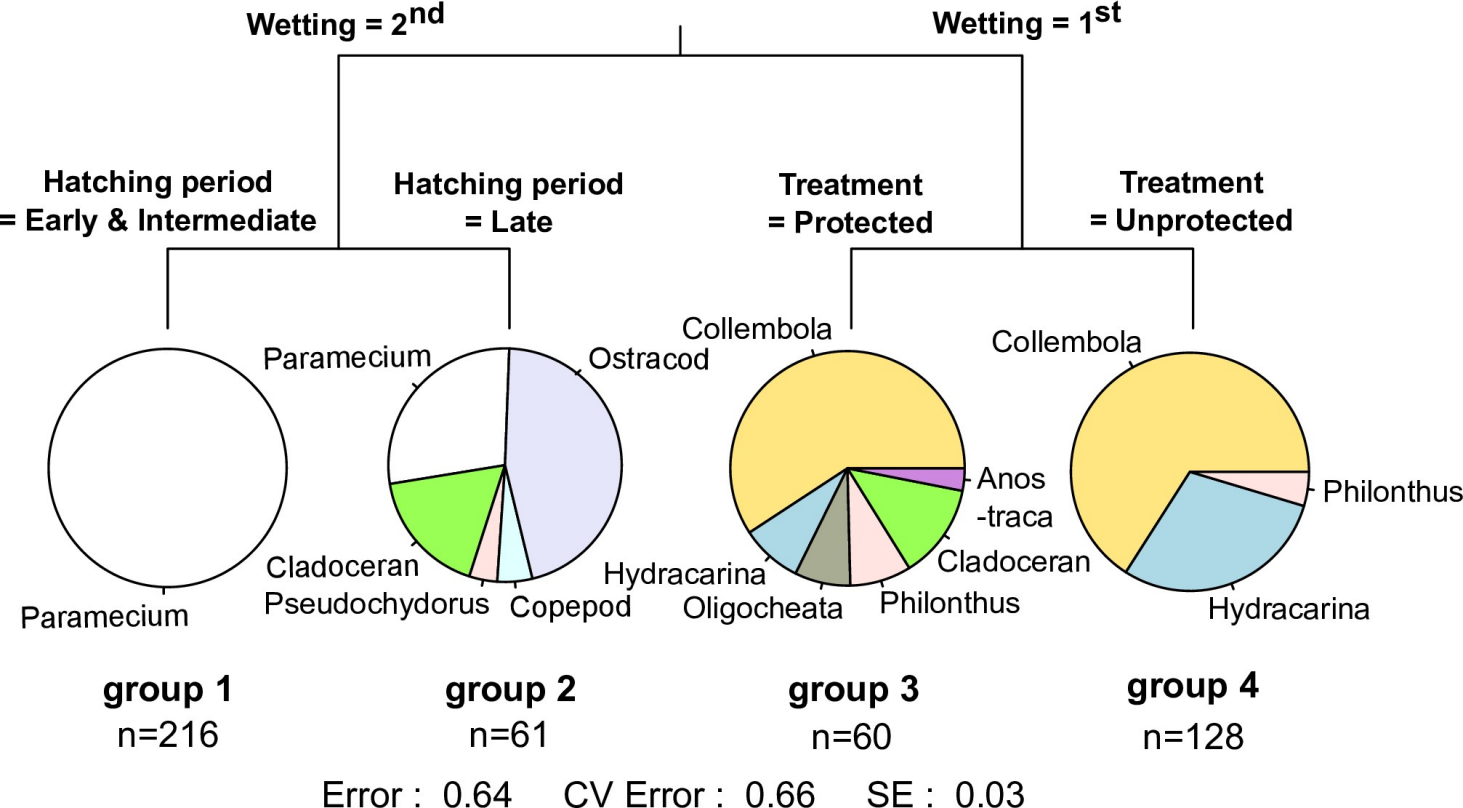
Multivariate regression tree (MRT) depicting the four different aquatic macroinvertebrate assemblages observed from hatching assays under conditions related to treatment (protected vs unprotected), inundation (1^st^ vs 2^nd^ inundation), hatching period (early, intermediate, late) from selected urban temporary wetlands in the City of Cape Town, Western Cape southern Africa. The pie charts at the terminal points depict the relative proportion of each species sampled for that particular set of treatment variables–species that contributed less than 5% were omitted.

The MRT indicated that the 1^st^ and 2^nd^ inundation periods were the primary differentiating split when describing aquatic macroinvertebrate composition (Fig 5). During the 2^nd^ inundation, the early and intermediate hatching period differed from the late hatching period. In contrast, the aquatic macroinvertebrate taxa emerging from the soil sediments in the 1^st^ inundation period differed between protected and unprotected wetlands. The number of aquatic macroinvertebrates observed during hatching assays was most influenced by inundation, with the second inundation producing higher abundances (Fig 5). Hatching period also influenced the number of aquatic macroinvertebrates observed, with the highest number seen in the early period, decreasing to the lowest number in the late period. Wetland type had little effect on the number of aquatic macroinvertebrates observed during hatching.

PERMANOVA revealed that aquatic macroinvertebrate assemblages emerging from the 1^st^ and 2^nd^ inundation were significantly different (PERMANOVA, pseudo-F = 13.34, p = 0.001), but overall aquatic macroinvertebrate assemblages from the protected and unprotected wetland sites were not different (PERMANOVA, pseudo-F = 1.25, p = 0.28) (Fig 5). During the aquatic wet phase sampling, collected aquatic macroinvertebrate assemblages from protected and unprotected wetlands were significantly different (PERMANOVA, pseudo-F = 2.54, p = 0.001), but between sampling periods there was no difference (PERMANOVA, pseudo-F = 1.35, p = 0.19) (Table 3). There is a possibility that spatial correlation is driving the differences in community assemblage in wet phase.

**Table 3:** PERMANOVA results comparing aquatic macroinvertebrate assemblage structure between the dry phase (hatching assays) and aquatic wet phase sampling. The dry phase compares aquatic macroinvertebrates composition between the two urban wetlands types and inundation, and the aquatic wet phase compares composition between the two urban wetlands types and during sampling occasions. Significant differences are marked with an asterisk.

Phase	Factors	Assemblage composition (response matrices)		
		df	F	P
Dry	Treatment (Protected vs Un-Protected)	1	1.25	0.28
	Wetting (1^st^ vs 2^nd^ inundation)	1	13.34	0.001*
Wet	Treatment (Protected vs Un-Protected)	1	2.54	0.001*
	Month (September vs October)	1	1.35	0.19

## Discussion

The increase in human migration into urban areas, like the City of Cape Town, together with landscape developments to support the growing populations, living spaces and service delivery have had deleterious impacts on the natural ecosystems and their biodiversity. Urban landscape developments have resulted in habitat loss and fragmentation causing some endemic terrestrial and aquatic flora and fauna to be at greater risk of being displaced [23, 42]. Investigating the impacts of urbanisation on aquatic macroinvertebrate communities within a matrix of protected versus unprotected urban temporary wetlands is an important aspect for the conservation and management of these systems [43, 44]. For example, Anderson [45], demonstrated that though protected and unprotected urban spaces can harbour similar terrestrial plant and insect patterns, management interventions can produce dramatic compositional shifts. Maintaining green space in urban areas is likely to provide a development strategy that will enhance urban biodiversity [23]. Temporary wetlands, despite providing many ecosystems services [3, 44], are faced with increasing threats from urbanisation. However, the impact of urbanisation on wetland aquatic macroinvertebrates, including the many rare and endemic species (i.e. amphipod, *P*. *pinnicornis*) recorded in these systems remains poorly documented [25, 29, 46].

Our hypothesis that unprotected urban wetlands will have lower aquatic macroinvertebrate diversity and exhibit significantly different community composition than protected urban wetlands was surprisingly not supported. Despite the differential human pressures between these wetlands types, aquatic macroinvertebrates hatching out from soil sediments collected during the dry phase largely showed no significant difference in all the three biodiversity measures employed; that is taxa richness, diversity (Shannon-Weiner) and evenness (Pielou’s). Similarly, univariate measure of aquatic macroinvertebrates collected during the wet phase had no significant differences between the urban wetland types. Average daily hatchlings and taxon accumulation also revealed the same pattern, of no significant differences, despite a greater variability in the unprotected urban wetlands. The only exception to this pattern was community composition, which showed a significant difference between these urban wetland types. In isolated, standing water bodies, like the wetlands or ponds studied in the current study, the length of hydroperiod has been demonstrated to positively affect biodiversity [27, 52]. Indeed, unprotected urban wetlands appeared to have a shorter hydroperiod, as they were sampled once in September and already dry in October, whereas protected urban wetlands seemed to have a slightly longer hydroperiod as they were sampled in both September and October. Predatory macroinvertebrates, e.g. dragonfly nymphs, aquatic bugs and calanoid copepods, are usually found in large numbers in wetlands with longer hydroperiod [25, 27], and in our study these predators were found exclusively in protected urban wetlands. However, the length of hydroperiod is predicted to positively affect community diversity [2, 8], but in our study, there was no significant difference between these urban wetlands with a variable hydroperiod. We can only speculate as to why this was the case, as the unprotected wetlands were clearly subjected to loitering, littering and dumping of rubbish as they are in a recreational area, while protected ones had little direct human interference. Perhaps, the impact of these direct human activities might not be as drastic as we initially thought or that the temporary wetland systems are resilient enough to withstand them.

The results of our study reveal similar patterns with those reported in the United Kingdom [19–21], but contrast those reported elsewhere [22, 23]. Bird and Day [47] concluded that a relatively narrow (∼100 m) buffer strip around the wetland is likely to be effective in the maintaining of natural conditions in terms of the physico-chemical water quality properties of a wetland. At the current level of human influence around these urban wetlands, it can be concluded from these results that, urbanisation impact is not readily detrimental to wetland aquatic macroinvertebrate communities. However, a bigger assessment including urban neighbourhoods of different socio-economic status is needed to test, at a bigger scale, the true universal validity of our current findings. It has been demonstrated that landscapes with lower urbanisation levels, which usually correlate well with the socio-economic status, can support higher species diversity, comparable to non-urban areas [48]. Further, some studies [22, 23] have reported little difference between urban and non-urban communities despite relatively high urbanisation levels. However, such observations do not take into account the sublethal effects of urbanisation, such a reduced growth, reproduction, etc [18].

The ecological differences that occur between the recurring wet and dry phases is a major challenge to making holistic management plans for temporary wetlands. Understanding the biotic elements, and therefore, the functioning, of these systems during the different phases, as well as the transition between these phases, provides an important baseline for the conservation of temporary wetlands. Understanding the hatching cycle of temporary wetland aquatic macroinvertebrates within their unique environmental context is essential for conservation. The fact that 70% of the total aquatic macroinvertebrate fauna found came from hatching of soil sediments highlights the important role of dormant or resting eggs in contributing to the biotic diversity of temporary wetlands and conservation of their fauna [2, 43, 44]. Interestingly, significant differences in diversity and evenness were observed between the 1^st^ and 2^nd^ wetting assays for protected wetlands, while unprotected wetlands showed significant differences in all diversity indices. This suggests that community structure is significantly influenced by the different processes, like bet-hedging of the eggs-bank [1, 31, 49]. Understanding which species emerge after a certain number of wettings and the mechanisms that govern this is vital for understanding the dynamics of aquatic macroinvertebrate propagules and has bearing on the wet phase as well. Species are likely to respond to successional wetting and drying as an environmental cue to emerge which supports the theory that resting stage of aquatic macroinvertebrates are heavily influenced by environmental conditions [50]. Aquatic macroinvertebrate communities in temporary wetlands are known to exhibit high levels of succession, with early “pioneers” radically different from the climax communities [51]. Hatching assays in this study confirmed this pattern. Typically, unicellular ciliates (i.e. Paramecium) and more insect species emerged during the early phases (i.e. Collembola and Coleoptera), while most microcrustacean taxa (i.e. Ostracods) emerged in the intermediate and late phases (Fig 5). The successional emergence of taxa suggests that different taxa have different environmental cues indicating favourable conditions [52].

The Western Cape province of South Africa, where the current study was conducted, experienced its worst drought in over a century between the years 2015–2017 [53]. Recent analysis of the responses of aquatic biodiversity demonstrated some unusual patterns, with some rare, endemic species that were not found on pre-drought sampling and are now occupying in low quality artificial habitats [54]. Drought could severely impact the community structure of temporary wetlands in the wet phase, as duration of the dry/wet phase changeover within temporary wetlands will influence which species emerge in the wet phase. Although, we could not sample the protected wetlands in October, as they were dry, it is surprising that aquatic macroinvertebrate composition between September and October samples within the protected wetlands were not dissimilar. Studies in the greater region have shown substantial changes in community composition between different inundation events and within each inundation [55]. The results of the current study will hence serve as baseline for comparison with post-drought years and/or high rainfall years.

## Conclusion

The urban landscapes are expanding at an alarming rate, and its ecological consequences are well documented and tend to be negative [22, 23]. The current study, even though based on a small sample size, demonstrated that unprotected urban wetlands can harbour same biodiversity of aquatic macroinvertebrate communities as protected urban wetlands. These results were unexpected, as we thought public access to unprotected urban wetlands would have drastically change their ecological communities. As such, these results present an opportunity to environmental managers of the city of Cape Town to prioritise these wetlands in their biodiversity planning and zoning scenarios, in order to afford these vulnerable ecosystems a chance of long-term protection from the worst impacts of urbanisation.

## Supporting information

S1 Appendix Dry phase. (CSV)

S2 Appendix Wet phase. (CSV)
